# Eptifibatide, an Older Therapeutic Peptide with New Indications: From Clinical Pharmacology to Everyday Clinical Practice

**DOI:** 10.3390/ijms24065446

**Published:** 2023-03-13

**Authors:** Gašper Tonin, Jasna Klen

**Affiliations:** 1 Faculty of Medicine, University of Ljubljana, 1000 Ljubljana, Slovenia; 2 Faculty of Arts, University of Ljubljana, 1000 Ljubljana, Slovenia; 3 Division of Surgery, Department of Abdominal Surgery, University Medical Centre Ljubljana, 1000 Ljubljana, Slovenia

**Keywords:** therapeutic peptides, glycoprotein IIb/IIIa inhibitors, antiplatelet drug, acute coronary syndrome, percutaneous coronary intervention, ischemic stroke, cardiogenic shock, carotid stenting, intracranial aneurysm stenting, septic shock

## Abstract

Therapeutic peptides are oligomers or short polymers of amino acids used for various medical purposes. Peptide-based treatments have evolved considerably due to new technologies, stimulating new research interests. They have been shown to be beneficial in a variety of therapeutic applications, notably in the treatment of cardiovascular disorders such as acute coronary syndrome (ACS). ACS is characterized by coronary artery wall damage and consequent formation of an intraluminal thrombus obstructing one or more coronary arteries, leading to unstable angina, non-ST elevated myocardial infarction, and ST-elevated myocardial infarction. One of the promising peptide drugs in the treatment of these pathologies is eptifibatide, a synthetic heptapeptide derived from rattlesnake venom. Eptifibatide is a glycoprotein IIb/IIIa inhibitor that blocks different pathways in platelet activation and aggregation. In this narrative review, we summarized the current evidence on the mechanism of action, clinical pharmacology, and applications of eptifibatide in cardiology. Additionally, we illustrated its possible broader usage with new indications, including ischemic stroke, carotid stenting, intracranial aneurysm stenting, and septic shock. Further research is, however, required to fully evaluate the role of eptifibatide in these pathologies, independently and in comparison to other medications.

## 1. Introduction

Peptide-based therapies are an emerging class of medications [1,2]. Following the rapid evolution of cutting-edge production, modification, and analytical technologies, peptide medication development has advanced significantly in recent years. These technological developments have helped to reduce the inherent limitations of peptides. As a result, a wide variety of natural and engineered peptides have been created, studied, and used for a range of medicinal purposes [3,4,5,6,7,8,9,10,11,12,13,14]. In the last two decades, more than 25 peptide drugs have been approved for clinical use, and over 150 peptides are in active development today [4,5].

Therapeutic peptides are notably utilized in the treatment of cardiovascular diseases, which are the leading cause of death and morbidity among non-communicable diseases [15]. Several important peptide drugs have been discovered in this field, primarily targeting hypertension, vascular function, and manifestations of coronary artery disease, such as acute coronary syndromes (ACS) [16,17,18,19].

In this narrative review, we focus on eptifibatide, an anti-aggregation peptide drug that targets the pathophysiological mechanism of platelet thrombus formation. First, the role of platelets in hemostasis and the function of the platelet glycoprotein IIb/IIIa (GPIIb/IIIa) receptor targeted by eptifibatide are presented. This is followed by a focused discussion on eptifibatide as a peptide drug, covering its pharmacological properties, clinical use, adverse effects, and interactions.

## 2. Hemostasis and the Role of the Platelets

Hemostasis is a physiological process involving the formation of a blood clot to halt bleeding. Adequate functioning of hemostasis is also responsible for ensuring blood flow [20,21]. Hemostasis can be explained as a three-step process:

- Vasoconstriction of the damaged vessel immediately after the injury to reduce blood loss;
- Adhesion, activation, and aggregation of platelets that form a platelet plug (usually referred to as primary hemostasis);
- Coagulation of protein factors and formation of a dense fibrin mesh (usually referred to as secondary hemostasis).

These processes are not necessarily consecutive steps but rather concurrent mechanisms that potentiate each other [21].

Platelets are short-lived (8−10 days) cell fragments originating from megakaryocytes. They play an essential role in hemostasis, which is most prominent in but not limited to primary hemostasis. The latter consists of platelet adhesion that starts with endothelial dysfunction and subendothelial matrix exposure. Different intracellular, membrane, and extracellular receptors and proteins mediate slowing and adhesion at the site of the defect. This process facilitates specific membrane and intracellular platelet changes, known as platelet activation. Platelets change shape and release their cytoplasmic granules with various activation factors that further promote adhesion, activation, and aggregation. They adhere to each other and extracellular molecules, including the proteins involved in the coagulation cascade. Consequently, they form a thrombus that arrests bleeding. Nevertheless, dysfunction of hemostasis and thrombus formation in diseases can also unnecessarily occlude the vessel and cause tissue ischemia [20,21,22,23].

## 3. Antiplatelet Medications

Antiplatelet medications influence primary hemostasis through their effect on platelets [21]. They prevent stent thrombosis, lessen arterial thrombosis following plaque degradation and rupture, and are used to treat and prevent myocardial infarction and ischemic stroke. There are numerous antiplatelet medications with various modes of action. Drugs with antiplatelet effects target the von Willebrand factor (VWF) interaction with glycoprotein Ib, glycoprotein VI (GPVI), the thrombin receptor PAR-1, the adenosine diphosphate (ADP) receptor P2Y12, cyclooxygenase, phosphodiesterase, or integrin receptor GPIIb/IIIa [24,25,26]. In Table 1, we provide a comparison between eptifibatide and other antiplatelet drugs with similar indications [27,28,29,30,31,32,33,34,35,36,37,38].

## 4. Receptor GPIIb/IIIa

GPIIb/IIIa, also called integrin αIIbβ3, is a receptor expressed on platelets and megakaryocytes. It plays a crucial role in platelet function, being mainly involved in the processes of primary hemostasis. Biochemically, GPIIb/IIIa is an integrin molecule, a type of transmembrane glycoprotein that can convey information both into (outside-in pathway) and out of (inside-out pathway) the cell. Integrin is a protein formed by two noncovalently bound subunits (α and β) and contains an extracellular domain, a transmembrane domain, and a small cytoplasmic tail [39,40]. Human platelets contain only two subgroups of integrins, namely integrins with subunits β1 and β3. However, there are more subgroups in the tissues of other mammals. Integrins in the platelets support their adhesion to the extracellular matrix molecules. GPIIb/IIIa is the primary integrin of platelets that can bind to ligands containing the arginine-glycine-aspartic acid sequence (such ligands are fibrinogen, fibrin, fibronectin, and the von Willebrand factor). As GPIIb/IIIa allows platelets to bind to its main ligand, fibrinogen, it cross-links them, enabling platelet aggregation. Loss of GPIIb/IIIa function is associated with a rare autosomal bleeding disorder, Glanzmann’s thrombasthenia, whereas its excessive activity is linked to arterial thrombosis [40,41,42,43]. Signaling of GPIIb/IIIa also plays a vital role in releasing vascular endothelial growth factors from platelets and cancer progression in tumor cells [40,44].

### 4.1. Receptor GPIIb/IIIa Pathway

As noted above, GPIIb/IIIa signal transmission is bidirectional. Accordingly, both the inside-out and outside-in pathways are discussed, as eptifibatide affects both when inhibiting GPIIb/IIIa.

#### 4.1.1. Inside-Out Pathway

The inside-out signaling pathway is initiated by the activation of G protein-coupled receptors by soluble agonists, such as ADP, epinephrine, thromboxane A2, and thrombin, or by the activation of other receptors by immobile agonists such as vWF and collagen. The inside-out pathway consists of several steps involving intracellular protein mediators such as talin and kindlin. It results in a conformational change of the extracellular domain that increases GPIIb/IIIa’s binding affinity for ligands, particularly fibrinogen [45].

In addition to intracellular activators talin and kindlin, other proteins may be involved in the activation of inside-out activation of GPIIb/IIIa. On the other hand, several proteins, such as docking protein 1 (Dok1), tensin 1, and filamin, are hypothesized to inhibit GPIIb/IIIa activation [40].

#### 4.1.2. Outside-in Pathway

The other part of bidirectional signaling is the outside-in pathway, which begins with the binding of fibrinogen to the activated GPIIb/IIIa. The main results are activation of adhesion, reorganization of the cytoskeleton, spreading of cells, and irreversible aggregation of platelets, leading to thrombus growth. Many proteins participate in the outside-in pathway, including transmembrane proteins, Rho-family small GTPases, intracellular adaptor molecules, kinases, and phosphatases. Most of them are associated with Src kinase activation, and the pathway often culminates in novel protein-actin cytoskeleton linkages and activities that promote primary hemostasis [40].

**Figure 1:**
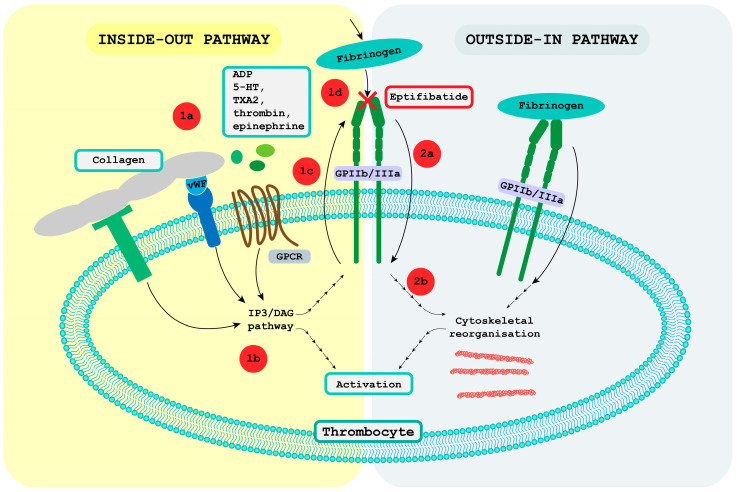
Bidirectional glycoprotein IIb/IIIa (GPIIb/IIIa) signal transmission. Numbers 1 and 2 present the steps of the inside-out and outside-in pathways, respectively. 1a: The activation of various receptors initiates inside-out signaling. G protein-coupled receptors (GPCR) are activated by soluble agents, namely adenosine diphosphate (ADP), 5-hydroxytryptamine (5-HT), thromboxane A2 (TXA2), thrombin, and epinephrine; other receptors are activated by collagen or the von Willebrand factor and collagen. 1b: Activation of these receptors leads to the initiation of many intracellular signaling pathways, the most dominant being the inositol trisphosphate (IP3)/ diacylglycerol (DAG) signaling pathway. The activated pathways result in platelet activation and a conformational change in the GPIIb/IIIa receptor. 1c: Protein mediators that interact with the intracellular domain of the GPIIb/IIIa receptor cause a conformational shift in the extracellular domain, increasing its binding affinity for ligands, particularly fibrinogen. 2a: Binding of ligand to GPIIb/IIIa receptor causes the activation of intracellular pathways. 2b: These pathways lead to platelet activation processes and novel protein-actin cytoskeleton linkages that cause cytoskeletal changes. As eptifibatide targets the GPIIb/IIIa receptor, it inhibits both pathways (highlighted by the red cross sign) [40,45].

Both pathways and the effect of eptifibatide on them are presented in Figure 1.

## 5. Eptifibatide

Eptifibatide (also known as Integrilin, Intrifiban, SB-1, or Sch-60936; DrugBank accession number: DB00063) is a heptapeptide derived from a disintegrin protein in the rattlesnake venom. It reversibly inhibits the GPIIb/IIIa, preventing platelet aggregation and activation [40,46,47,48].

### 5.1. Associated Drug Classes

Eptifibatide belongs functionally to the GPIIb/IIIa inhibitors (GPI) and, with respect to its structure and origin, to the family of disintegrin proteins [46]. This section briefly presents the pharmacological basis of eptifibatide classification.

#### 5.1.1. GPIIb/IIIa Inhibitors

Several drugs affect GPIIb/IIIa, but three GPI are the most prominent, namely eptifibatide (Integrilin^®^), tirofiban (Aggrastat^®^), and abciximab (ReoPro^®^), which are all administered intravenously. In addition, some active oral peptide agents targeting GPIIb/IIIa, such as orbofiban, xemilofiban, sibrafiban, and roxifiban, have been tested. However, these have not shown encouraging outcomes and have been linked to an increase in mortality, warranting the termination of many clinical trials [40,49,50,51,52,53,54,55].

#### 5.1.2. Snake Venom and Disintegrin Peptide Family

With several derivative drugs in clinical or research use, snake venoms are an attractive natural source for drug discovery and development [56,57]. They consist of a wide array of molecules, most of which are bioactive and have toxic effects on muscles, neurons, the heart, or other organ cells. Nevertheless, snake venom has been employed in medicine since ancient times, notably in traditional Chinese medicine. Moreover, in the 17th century, the Italian Felice Fontana demonstrated the effect of snake venom on human blood [58]. Several toxins are now recognized as valuable therapeutic agents or diagnostic tools [47,56]. The United States Food and Drug Administration (FDA) has already approved numerous snake venom-derived drugs, including Integrilin^®^ (eptifibatide), Captopril^®^ (enalapril), Aggrastat^®^ (tirofiban), Reptilase^®^ (batroxobin), and Exanta^®^ (ximelagatran). In addition, many medications are now at the preclinical and clinical stages of testing for therapeutic use [47].

Pharmacologically, snake venom consists of various substances with numerous effects. Some of them are peptides that mainly target (in an enzymatic or non-enzymatic way) membrane receptors, enzymes, ion channels, and elements of the hemostatic system [47].

Disintegrins are a class of small cysteine-rich peptides that target integrins and are found in different species of snake venom. They carry the KTS, MGD, RTS, VGD, KGD, WGD, or RGD amino acid motifs recognized by integrins. It is important to understand that proper cysteine bridges are essential for protein folding and conformational exposure of the binding motif, composed of three amino acids. As different motif exposure translates to different effects on different integrin protein types, these short peptides are involved in several processes. For example, they take part in the regulation of angiogenesis, platelet aggregation, apoptosis, cell migration, invasion, adhesion, and proliferation [47,57].

Disintegrins may be classified into four groups [57]:

- Approximately 41–51 amino acid long peptides with four cysteine bridges (echistatin and obtustatin);
- Approximately 70 amino acid long peptides with six cysteine bridges (barbourin, flavoviridin, and atrolysin);
- Approximately 84 amino acid long peptides with seven cysteine bridges (bitistatin);
- Macromolecular complexes of usually noncovalently bound homodimers or heterodimers, which are 67 amino acids long and have 10 cysteines incorporated into the structure.

Eptifibatide is a disintegrin type of peptide that mimics a portion of barbourin, a toxic peptide found in the venom of the Southeastern pygmy rattlesnake (*Sistrurus miliarius barbouri*) [57]. Barbourin is selective for GPIIb/IIIa, despite other disintegrins having nonspecific affinities for different integrins. This nonspecific binding of other disintegrins is mediated by the RGD motif, whereas barbourin derives its favorable selectivity from the unique KGD motif in which lysine is replaced by arginine. In eptifibatide, the KGD motif is preserved but modified [59]. In the Protein Data Bank (PDB), there are currently three models of the GPIIb/IIIa complexes with eptifibatide (PDB ID: 7THO, 2VDN, and 7U60) [60,61,62].

### 5.2. Biochemical Structure

**Figure 2:**
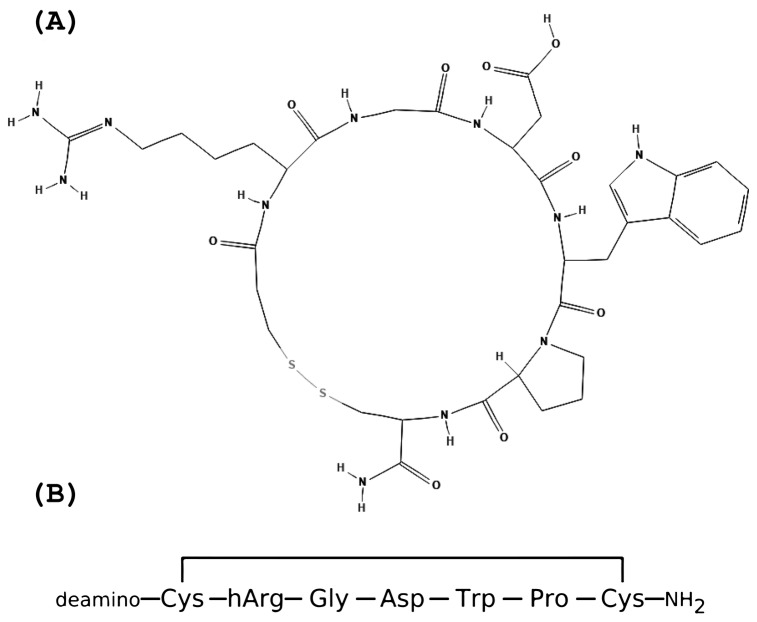
We present eptifibatide’s structure and amino acid sequence. Both were obtained from PubChem [63]. (**A**) Eptifibatide structure; (**B**) Eptifibatide amino acids sequence.

Eptifibatide is a cyclic peptide derived from the disintegrin family protein, barbourin [57]. Its molecular formula is C35H49N11O9S2, and its molecular mass is 832.0 g/mol [63]. Both the structure and the amino acids sequence are presented in Figure 2.

It was derived by determining the minimum active sequence (MAS) of this component of snake venom. A minimum active sequence (MAS) is the shortest amino acid sequence derived from an endogenous peptide, still retaining its potency or binding affinity to its target [46]. The process of truncation is used to remove biologically redundant amino acid residues from the protein. The endogenous peptides are “trimmed” in such a way as to reach a more economical protein size that can be widely synthesized without compromising its effect on biological targets. The cys-rich endogenous disintegrin protein from snake venom contains 73 amino acids (UniProt code: P22827), and a length of seven amino acids was achieved for eptifibatide by the process of truncation [46,59,64]. The small size of the peptide explains its low immunogenicity, which is an essential factor in repetitive administration and the use of the drug on patients with an unknown history [59].

Moreover, in developing eptifibatide, researchers had to overcome the drawbacks of peptide drugs, especially their low in vivo stability and membrane impermeability [3]. Since the direct action of eptifibatide is thought to be limited only to the extracellular domain of GPIIb/IIIa on platelets, the drawback of membrane impermeability was eliminated. To increase the stability of eptifibatide, the peptide was cyclized using a disulfide bridge between the captopropionyl residue (des-amino-cysteinyl) and the cysteine. The cyclic structure increases the bioavailability of the drug and its resistance to plasma proteases [59]. Besides these modifications, the peptide undergoes guanylation at the Lys side chain and deamination at the N-terminus (among other modifications), giving it a highly potent therapeutic value [46,65].

### 5.3. Pharmacodynamics

Eptifibatide competes in a dose-dependent manner with fibrinogen for the GPIIb/IIIa. It is a specific inhibitor of the GPIIb/IIIa receptor, which limits the pharmacological effect of platelets and their precursors [59]. The treatment objective is to achieve 80% inhibition of platelet aggregation depending on the dose and concentration of medication. This proposition has been demonstrated ex vivo with adenosine diphosphate (ADP) and other agonists that induce platelet aggregation. The immediate effect of eptifibatide can be observed after an intravenous injection; when a continuous infusion is subsequently administered, this treatment can successfully inhibit more than 80% of ADP-induced platelet aggregation ex vivo with normal calcium levels in the majority of patients [66]. Furthermore, the eptifibatide effect can be quickly stopped, since the drug rapidly dissociates from GPIIb/IIIa, and after 4 h, platelet functions return to baseline and are swiftly cleared from plasma [59].

### 5.4. Pharmacokinetics

Intravenous administration of therapeutic peptides has the advantage of avoiding pre-systemic metabolism by the liver and gastrointestinal enzymes, resulting in complete systemic availability. For bolus doses of 90 to 250 µg/kg and infusion rates of 0.5 to 3.0 µg/min, the pharmacokinetics of eptifibatide are linear and proportional to the dose. When infused at 2.0 µg/kg/min, the mean steady-state plasma concentration of eptifibatide in patients with coronary artery disease is 1.5 to 2.2 µg/mL. Plasma concentrations in this range can be attained rapidly if a bolus of 180 µg/kg is used prior to the infusion [66]. The onset of action is rapid, with inhibition of platelet aggregation occurring 15 min after a bolus. The binding proportion of eptifibatide to human plasma proteins is approximately 25% [66].

The pharmacokinetics of peptides are characterized by their typically short half-life in the bloodstream, which results from cleavage by proteases and peptidases. A short elimination half-life for endogenous peptides is desirable for regulating their concentrations and function. The eptifibatide plasma elimination half-life is 2.5 h [2]. Peptides have a molecular weight between 1 and 10 kDa; therefore, the primary absorption process is diffusion-driven uptake into blood. On the other hand, eptifibatide has a molecular weight of 800 D. The volume of distribution for eptifibatide is 0.2 to 0.3 L/kg.

Eptifibatide is not known to be metabolized by uridine-5-diphosphate glucuronosyltransferase enzymes or cytochrome P450 (CYP) but is deaminated by metabolic enzymes. Furthermore, kidney clearance accounts for approximately 50% of total body clearance; therefore, deaminated eptifibatide and polar metabolites are excreted in the urine [66]. Hepatic metabolism is not the primary route of elimination for most peptides, but it can play an essential role in the metabolism of some peptide drugs [2].

### 5.5. Clinical Applications

Eptifibatide is an antiplatelet agent; therefore, it is used in diseases in which thrombus formation is a critical part of pathogenesis or complications. As with any antithrombotic treatment, consideration should be given to the trade-off between the risk of ischemic injury and bleeding when administering eptifibatide [67]. The FDA indicates its use for the treatment of ACS and in percutaneous coronary intervention (PCI) [68]. However, research over the past decade has also sought to evaluate the role of eptifibatide in ischemic stroke, stenting of carotid and intracranial aneurysms, and septic shock [68,69,70,71,72,73]. In Table 2 we present summary of recently published meta-analyses.

#### 5.5.1. Acute Coronary Syndromes: Angina Pectoris, STEMI, and NSTEMI

ACS is a manifestation of coronary heart disease associated with an abrupt reduction in the blood supply to the heart. Underlying factors contributing to the disease are smoking, hyperlipidemia, obesity, diabetes, etc. The syndromes comprise different clinical presentations, including unstable angina pectoris, non-ST elevation myocardial infarction (NSTEMI), and ST-elevation myocardial infarction (STEMI). Despite different presentations, all syndromes usually present with chest discomfort at rest [78,79,80]. In most cases, the pathophysiological basis of these diseases is the rupture of an atherosclerotic plaque in a cardiac vessel causing platelet aggregation and thrombus formation, which in turn restricts the blood flow to the heart tissue, resulting in cardiac ischemia [78,79]. 

##### Angina Pectoris and non-ST Elevation Myocardial Infarction

Eptifibatide is indicated for the prevention of myocardial infarction in unstable angina and NSTEMI in both drug-treated and PCI patients [68,81]. The PURSUIT trial (1998) showed a reduction in endpoint mortality and a beneficial effect in preventing nonfatal myocardial infarction in these patients [81]. In NSTEMI, a combination of loading dose by aspirin and maintenance treatment by eptifibatide could be used [67]. Aspirin inhibits thromboxane A2 production and therefore prevents platelet aggregation. Although both drugs affect the platelets, their mechanisms of work are different, potentiating their effect.

##### ST-Elevated Myocardial Infarction

A meta-analysis by Karathanos et al. in 2019 showed that routine use of GPIs in patients with STEMI was associated with reduced mortality, which was probably the consequence of a reduction in recurrent ischemic events. Despite the promising result, it should be noted that these results are largely based on studies before dual antiplatelet therapy with prasugrel/ticagrelor was routinely used, as is the case today [76]. Although less convincing, eptifibatide can also improve myocardial perfusion in STEMI, as shown in the TITAN-TIMI 34 trial [68,82]. Nevertheless, more recent studies have shown that prehospital administration of GPI in STEMI has not shown benefits and even increases the bleeding risk compared to routine use in a catheterization laboratory [83,84,85]. Although eptifibatide has not been tested in a randomized trial, the European Society of Cardiology guidelines (ESC) suggest it as bail-out therapy in high-risk patients (slow flow or no flow with occlusion of the stent, high thrombus burden, etc.) but not as a routine drug for primary PCI [85]. 

A meta-analysis by Saleiro et al. in 2020 has shown that the use of GPIs as an adjunct to standard therapy may be beneficial in myocardial infarction that results in cardiogenic shock. In this study, the use of GPIs was associated with better outcomes, namely short-term and long-term survival. Moreover, it did not increase the risk of bleeding in the treated patients [77]. Other newer studies have shown similar results [77,86,87].

#### 5.5.2. Percutaneous Coronary Intervention

PCI is a non-surgical but invasive procedure in which a catheter is used to insert a stent into narrowed or occluded coronary arteries, improving the blood supply. It is the preferred method of treatment for ACS [88,89]. As the pretreatment in patients undergoing PCI, the ESC guidelines propose using a combination of eptifibatide and unfractionated heparin, as both anticoagulation and platelet inhibition are important in the pathogenesis-based therapy of NSTEMI [67]. Guidelines from the American College of Cardiology (ACC) and the American Heart Association (AHA) similarly suggest the use of eptifibatide as an initial antiplatelet therapy in patients with high-risk features [90]. Heparin dosages of 50–70 IU/kg i.v. should be used if administered in this combination [67].

Although the FDA has approved using eptifibatide for patients undergoing PCI (including stenting), data for using eptifibatide in the periinterventional treatment of NSTEMI are limited and partially outdated. A major trial that has shown the benefits of eptifibatide use in PCI was the IMPACT-II trial, published in 1997 [68,91]. Most research on the use of eptifibatide in PCI pretreatment predates routine dual antiplatelet treatment (DAPT) [92,93,94]. In periinterventional antiplatelet treatment, oral P2Y12 receptor inhibitors were found to be as effective as GPI and are recommended for routine use [67,68,92,93,94]. Therefore, little to no evidence exists to support the use of eptifibatide in patients who will undergo coronary angiography and are receiving DAPT [92,93,94]. On the other hand, eptifibatide may be considered when facing high-risk PCI patients (slow flow or no-flow with the closure of the stent, high thrombus burden, etc.), patients who did not receive pretreatment with P2Y12 receptor inhibitors, and patients with thrombotic complications [67,94,95]. The intracoronary administration of the drug is comparable to intravenous use [96,97].

#### 5.5.3. Bridging Strategy for Patients Undergoing Surgery after Coronary Stent Insertion

Postoperative bleeding prevention after cardiac surgery is crucial to decreasing morbidity and mortality. Since i.v. antiplatelet medications, such as eptifibatide, are quickly cleared from the system and their antiplatelet effect can be quickly reversed, they are used before cardiac and noncardiac surgery as a substitution for oral P2Y12 inhibitors [98]. A 2022 meta-analysis by Wu et al. showed that eptifibatide might be safe and effective when used as a bridging strategy for patients undergoing coronary stent implantation requiring surgery. The GPI might be used without an increased bleeding risk when temporarily discontinuing DAPT. Nevertheless, further randomized studies are needed to substantiate this claim [75,99]. In a 2019 study by Van Tuyl et al., eptifibatide was shown to be an effective choice for these patients and was even preferred over abciximab [98].

Another drug that is often compared to eptifibatide in bridging strategies is cangrelor. Cangrelor is a reversible P2Y12 receptor inhibitor that prevents ADP-induced platelet aggregation and activation. It should be considered in patients with renal insufficiency, as clearance of eptifibatide is influenced by renal function [98]. Moreover, a study by Yun et al. from 2019 showed that cangrelor and eptifibatide were similar in terms of overall bleeding events and major inpatient cardiac adverse events [100].

#### 5.5.4. Ischemic Stroke and Carotid and Intracranial Aneurysm Stenting

The CLEAR trial from 2008 showed that eptifibatide is beneficial in preventing intracerebral hemorrhage in patients with acute ischemic stroke if administered with TPA [68,68,69]. Nevertheless, a 2022 meta-analysis by Liu et al. showed that adding eptifibatide to routine thrombolysis or thrombectomy treatment did not improve functional outcomes, favorable outcomes, or the National Institutes of Health Stroke Scale (NIHSS) score. Moreover, it might be associated with an increase in fatal ICH three months after AIS [74]. Another meta-analysis by Zhu et al. in 2020 showed that eptifibatide might be promising when used at a reduced dose (a low dose was also used in the CLEAR study); thus, more randomized trials with different doses are needed to evaluate the role of eptifibatide in the treatment of acute ischemic stroke [68,69,70]. A newer retrospective case-control study by Luo et al. (2022) compared routine therapy and treatment with an additional low dose of eptifibatide. Although the study reported no significant differences in NIHSS or adverse events, an analysis of the subgroups showed that eptifibatide is a safe and effective treatment when small artery occlusion is involved [71]. Similarly, a trial by Rana et al. from 2022 showed that using eptifibatide during endovascular therapy in large vessel occlusion is associated with a higher rate of hemorrhages and no benefits to the NIHSS or 90-day mortality [101]. On the other hand, a matched-control analysis by Ma et al. in 2022 showed that the use of eptifibatide was safe and effective in patients undergoing mechanical thrombectomy after ischemic stroke, because the rate of successful recanalization was significantly higher in the intervention group (91.3% versus 81.5%; *p* = 0.043) and the 3-month outcome on the modified Rankin Scale showed good results (53.1% versus 33.3%; *p* = 0.016) [102]. As shown, the existing evidence in this research area is not yet conclusive, and further studies are needed to evaluate the use of eptifibatide in the treatment of ischemic stroke.

Antiplatelet agents are administered to prevent one of the most critical complications of stenting, namely stent thrombosis. In a study by Osteraas et al., the use of eptifibatide (as a bolus followed by infusion for 24 h after stent placement) was shown to be associated with a lower risk of symptomatic intracranial hemorrhage after carotid stenting [103]. Another study by Horev et al. from 2021 similarly reported a reduced number of complications when using eptifibatide immediately after carotid stenting [104].

Eptifibatide has also been explored as a potential antiplatelet therapy in the stenting of intracranial aneurysms. A 2022 study by Aouni et al. compared three antiplatelet agents (ticagrelor, eptifibatide, and cangrelor) in the stent-assisted endovascular treatment of unruptured intracranial aneurysms and found no significant differences between them [73].

#### 5.5.5. Septic Shock

One of the main mechanisms in septic shock is the activation of the endothelium and platelets. This activation subsequently leads to generalized microvascular damage, microthrombi, capillary leaks, and coagulopathy caused by widespread consumption of coagulation factors [72,105]. It is noteworthy that research on the use of eptifibatide in septic shock is extremely limited, as to our knowledge, only one study has been performed. The 2019 randomized and placebo-controlled double-blind trial by Berthelsen et al. showed the benefits in improving Sequential Organ Failure Assessment (SOFA) and reducing platelet consumption, fibrinolytic biomarkers, and endothelial damage when a combination of the synthetic analogs of prostacyclin, iloprost, and eptifibatide was used in patients with septic shock [72]. Further research in this area is needed to elucidate the role of eptifibatide.

### 5.6. Contraindications

Particular attention should be paid to patients who are hypersensitive to the active ingredient or any of the additives, as well as patients on any other parenteral GPI. Another group of contraindications that are important in the use of eptifibatide includes a history of bleeding diathesis within 30 days, current ongoing internal bleeding, and recent significant bleeding within the last six months, either in the gastrointestinal or genitourinary tract [68]. Furthermore, eptifibatide is not recommended in hemodialysis patients due to an increased risk of bleeding. An alternative choice for treatment is abciximab, a mouse-human monoclonal antibody based on a murine analog [106]. Similarly, severe, uncontrolled hypertension with a systolic blood pressure of >200 mmHg and a diastolic blood pressure of >110 mmHg in patients receiving antihypertensive treatment and a history of hemorrhagic stroke are also contraindications for the use of eptifibatide [68]. Other conditions where eptifibatide is contraindicated include thrombocytopenia with a platelet count of <100,000 cells/mm^3^ and a prothrombin time > >1.2 × higher from control values or international normalized ratio (INR) ≥ 2.0 [68]. Thrombocytopenia is frequently observed in individuals with hepatic dysfunction; this is thought to be related to splenic sequestration due to portal hypertension or decreased production of thrombopoietin by damaged liver cells. It is also known that bone marrow suppression or folic acid deficiency may occur in people with alcoholic cirrhosis. Accordingly, eptifibatide is contraindicated in these categories of patients [107]. We must be mindful of two other important conditions when initiating eptifibatide, namely recent major surgery or trauma within the past six weeks and a history of intracranial neoplasms, arteriovenous malformations, or aortic dissections.

In addition, eptifibatide is a category B drug during pregnancy; however, it should be used in ACS because the benefits outweigh the risks. Nevertheless, special caution is needed in lactating mothers. Caution is also advised for women, the elderly, and people with a low body weight [68].

### 5.7. Administration and Dosages

Eptifibatide is an effective periinterventional antiplatelet agent manufactured as a sterile solution in 10 mL single-dose vials containing 20 mg of eptifibatide and in 100 mL single-dose vials containing 75 mg of eptifibatide. It can be administered via IV and must be protected from direct light prior to administration [68]. Before administration, baseline blood tests, such as complete blood count, prothrombin time (PT)/activated partial thromboplastin time (aPTT), serum creatinine, and activated clotting time, should be performed in patients undergoing PCI. During administration, diligent assessment and monitoring for possible arrhythmias should be performed.

The dose regimen for NSTEMI is a double bolus of 180 μg/kg i.v. in 10 min intervals, followed by an infusion of 2.0 μg/kg/min for up to 18 h in patients with normal kidney function. Patients with a creatinine clearance of less than 50 mL/min should have a 50% reduction in their eptifibatide dose. In elderly patients, the hemostatic balance is shifted towards an increased tendency for clotting and reduced fibrinolysis. However, several other contributing factors exist in the elderly that can lead to an increased risk of bleeding. These include distinct pharmacokinetic and pharmacodynamic responses, polypharmacy, and increased comorbidities, all of which can interact with one another and increase the risk of bleeding. Nevertheless, while caution should be exercised in the elderly, advanced age is not a contraindication for the use of GPI [108].

Eptifibatide may be administered concurrently with heparin and aspirin, as it was used in all clinical studies, and the dosage need not be altered. It is known that the concentration of eptifibatide in plasma increased rapidly and remained at a constant level of about 1640 ng/mL from half an hour to 24 h. After that, the concentration of the drug dropped rapidly in multiple stages [66,67].

### 5.8. Adverse Effects and Interactions

#### 5.8.1. Adverse Effects

Eptifibatide may cause a rare and significant adverse effect known as eptifibatide-induced thrombocytopenia (EIT), characterized by an acute, unexpected decrease in platelet count to less than <30,000 platelets/µL. However, patients must be exposed to eptifibatide for 5–7 days before they develop sensitization when receiving it for the first time. EIT was reported in several case studies and occurred in approximately 1 to 2% of patients [109,110,111,112,113,114].

It is essential to monitor platelets frequently after starting treatment. The underlying causes of EIT are not fully understood; however, research indicates that it may involve the formation of IgG antibodies that target GPI only when the implicated drug is present. IgG can cause platelet aggregation and secretion, along with the activation of the tyrosine kinase cascade [115]. Enzyme-linked immunosorbent assay testing using monoclonal antibodies can identify platelet glycoprotein targets, GPIIb/IIIa; however, such testing is not widely accessible [68,116]. Treatment options for EIT include stopping the infusion, platelet transfusions (which may be inefficient because of the half-life of GPI), and fresh frozen plasma (used in major bleeding). Other treatment options include the use of corticosteroids (which are not effective) and intravenous immunoglobulin G (IVIG) [117]. 

Bleeding is a more common and potentially serious adverse effect. Masood et al. showed that out of 28 reported complications of EIT, 57% involved bleeding events. These included 14% catheterization site bleeding, 14% petechial hemorrhage, 7% groin hematoma, 7% infusion site bleeding, 3.6% gastrointestinal bleeding, 3.6% hemoptysis, 3.6% epistaxis, and 3.6% hematuria [117]. Diffuse alveolar hemorrhage (DAH) is another important but often underappreciated form of eptifibatide-related bleeding. The incidence rate of eptifibatide-induced DAH is 0.5% [118].

In addition to the bleeding and thrombocytopenia mentioned above, side effects on the heart should not be forgotten. The PURSUIT study reported various arrhythmias, such as ventricular tachycardia, atrial fibrillation, ventricular fibrillation, and atrioventricular block, in addition to congestive heart failure and cardiac arrest. However, the side effects were attributed to the underlying disease [81].

As for the other side effects, it is worth mentioning thrombosis, which occurred in 10.75% of patients and included deep vein thrombosis, pulmonary embolism, and in-stent thrombosis. However, the exact relationship between eptifibatide and thrombosis is unclear. The following side effect also occurred in some proportion, namely allergic reactions such as rigor, chills, angioedema, and hypotension. Positive eptifibatide antibodies were also found in 87.5% of cases [81,117].

#### 5.8.2. Interactions

There are 140 drugs known to interact with eptifibatide, of which 55 had major interactions. Considering the mechanism of action of eptifibatide, caution should be exercised with drugs inhibiting platelet aggregation. These drugs include clopidogrel, ticlopidine, thrombolytics, oral anticoagulants, adenosine, prostacyclin, sulfinpyrazone, nonsteroidal anti-inflammatory drugs (NSAIDs), dipyridamole, and dextran solution. On the other hand, if an oral anticoagulant such as warfarin was taken concomitantly, no further bleeding was observed [68]. 

## 6. Conclusions

In conclusion, eptifibatide is a useful cardiac peptide drug that is slowly gaining value in new indications such as the treatment of ischemic stroke, carotid stenting, stenting of intracranial aneurysms, and septic shock. However, more extensive randomized trials are needed to confirm its therapeutic value in these conditions.

## Figures and Tables

**Table 1:** Comparison of different antiplatelet drugs [27,28,29,30,31,32,33,34,35,36,37,38].

Drug	Eptifibatide	Prasugrel	Ticagrelor	Cangrelor	Abciximab
Indication	acute coronary syndrome (ACS)	ACS	ACS	ACS	ACS
Main Contraindications	active bleeding, thrombocytopenia, severe, uncontrolled hypertension, major surgery or trauma within the prior six weeks, renal failure	active bleeding, cerebrovascular insult (CVI), transient ischemic attack (TIA), severe liver failure	active bleeding, concomitant use of ticagrelor with strong CYP3A4 inhibitors, severe liver failure	active bleeding, CVI, TIA	active bleeding, thrombocytopenia, vasculitis, severe uncontrolled hypertension, intracranial tumour, major surgery or trauma in the last six weeks
Drug Type and Mechanisms	glycoprotein IIb/IIIa receptor inhibitor	thienopyridine prodrug, an irreversible antagonist of the ADP P2Y12-receptor	cyclopentyl-triazolo-pyrimidine drug, direct-acting P2Y12-receptor antagonist	adenosine triphosphateanalogue, reversibly binds to P2Y12-receptor	glycoprotein IIb/IIIa receptor inhibitor
Time to Peak Action	15 min	30 min	1.5 h	2 min	30 min
Half-Life	2 h	7 h	7 h	3–6 min	30 min
Dose Administration	a double bolus of 180 μg/kg i.v. in 10 min, followed by an infusion of 2.0 μg/kg/min for up to 18 h	loading dose 60 mg, maintenance dose 10 mg/day	loading dose 180 mg, maintenance dose 90 mg/12 h	bolus 30 μg/kg injection then 4 μg/kg/min infusion	bolus 0.25 mg/kg injection then 0.125 μg/kg/min infusion for 12 h
Route/Dosing Interval	intravenous	oral, once daily	oral, twice daily	intravenous	intravenous
Major Side Effects	bleeding, thrombocytopenia	bleeding, thrombotic thrombocytopenic purpura, headache, dizziness	bleeding, dyspnea, hyperuricemia	bleeding, dyspnea	bleeding, chest pain, hypotension, injection site pain, abdominal pain, nausea, vomiting
Excretion	excreted in urine	~70% of the dose is excreted in urine and ~30% in the faeces, as inactive metabolites	metabolized in the liver	~60% of the dose is excreted in urine and ~35% in faeces	excreted in urine

**Table 2:** Summary of recently published meta-analyses on the clinical use of eptifibatide.

Authors	Research Design	Number of Studies and Patients	Results	Main Findings
Liu et al., 2022 [74]	Analysis of studies using tirofiban and eptifibatide in patients with acute ischemic stroke (AIS). Use of the modified Rankin scale (mRS) to evaluate favorable and functional outcomes.	Twelve studies (two randomized control trials and ten prospective cohort studies) with 2926 patients.	Treatment with tirofiban or eptifibatide might increase mortality (relative risk (RR) = 0.84, 95% confidence interval (CI) 0.71–0.99, *P* = 0.121) but had no effects on the favorable outcome (RR = 1.09, 95% CI 0.89–1.35, *p* = 0.411), functional outcome (RR = 1.12, 95% CI 0.98–1.28, *p* = 0.010), and last available NIHSS (WMD = −2.32, 95% CI −5.14 to 0.50, *p* = 0.106).	Adding tirofiban or eptifibatide to thrombolysis/thrombectomy was not significantly associated with a favorable outcome (mRS = 0–1) nor a functional outcome (mRS = 0–2) in patients with AIS at 3 months but might be associated with higher mortality, possibly due to fatal intracranial hemorrhage.
Wu et al., 2022 [75]	The use of glycoprotein IIb/IIIa inhibitors (GPI) in patients requiring surgery after coronary stent placement. The primary outcome was the success rate of no major adverse cardiovascular events, and the secondary outcome the success rate of no reoperations for bleeding management.	Ten studies with 382 patients (four studies with eptifibatide with 167 patients).	For the primary endpoint, the success rate was 97.7% (95% CI 94.4–98.0%) for GPI and 95.8% (95% CI 90.4–99.4%) for eptifibatide. For secondary endpoints, the success rate was 98.0% (95% CI 94.8–99.9%) for GPI and 95.3% (95% CI 88.5–99.4%) for eptifibatide.	GPI are safe and effective as a bridging strategy for patients that require surgery following recent stent implantation.
Zhu et al., 2020 [70]	Analysis of safety of GPI in AIS. The two main outcomes analyzed were RR of death and 90-day intracerebral hemorrhage (ICH).	Twenty studies with 3700 patients	The RR values of symptomatic ICH for abciximab and eptifibatide were 4.26 (1.89, 9.59) and 0.17 (0.04, 0.69).	Eptifibatide can be considered safe in low doses and if used in suitable patients. Its use showed no statistically significant effect on any ICH and death. The incidence of symptomatic ICH was reduced after the administration of eptifibatide.
Karathanos et al., 2019 [76]	The use of GPI in selected patients with ST-elevation myocardial infarction (STEMI).	Twenty-one studies with 8585 patients.	A significant reduction in all-cause mortality at 30 days (2.4% [GPI] vs. 3.2%; risk ratio RR = 0.72; *p* = 0.01) and 6 months (3.7% vs. 4.8%; RR = 0.76; *p* = 0.02), and a reduction in recurrent myocardial infarction (1.1% vs. 2.1%; RR = 0.55; *p* = 0.0006).	GPI administration in STEMI decreased mortality, although that was mostly shown in pre-prasugrel/ticagrelor studies. Trials utilizing modern STEMI treatment are required to validate these results.
Saleiro et al., 2020 [77]	Use of GPIs as an adjunctive therapy in myocardiac infarct, complicated by cardiogenic shock. Analyzed outcomes were mortality, angiographic success, and bleeding events.	Seven studies with 1216 patients.	A 45% relative reduction in the odds of death at 30 days (pooled OR 0.55; 95% CI 0.35–0.85; I2 = 57%; *p* = 0.007) and a 49% reduction in the odds of death at 1 year (pooled OR 0.51; 95% CI 0.32–0.82; I2 = 58%; *p* = 0.005). Increased probability of achieving better flow (pooled OR, 2.05; 95% CI 1.37–3.05; I2 = 37%, *p* = 0.0004). Major bleeding events were not increased with GPI therapy (pooled OR, 1.0; 95% CI 0.55–1.83; I2 = 1%, *p* = 0.99).	GPIs are efficient as adjunct therapy and are associated with better short-term and long-term survival. They also didn’t increase the risk of bleeding.

## Data Availability

Not applicable.
